# Integrative proteomic and metabonomic profiling elucidates amino acid and lipid metabolism disorder in CA-MRSA-infected breast abscesses

**DOI:** 10.3389/fcimb.2023.1240743

**Published:** 2023-11-13

**Authors:** Yongshuo Yin, Lina Cao, Meng Zhang, Yingjie Li, Chunhua Sun, Qinghua Ma, Zhaoyun Liu, Chao Li, Zhiyong Yu, Xiao Guan

**Affiliations:** ^1^ Department of Breast Surgery, Shandong Cancer Hospital and Institute, Shandong First Medical University and Shandong Academy of Medical Sciences, Jinan, Shandong, China; ^2^ Department of Breast Surgery, Shandong University Cancer Center, Jinan, Shandong, China; ^3^ Department of Health Management Center, Qilu Hospital, Cheeloo College of Medicine, Shandong University, Jinan, Shandong, China; ^4^ Department of Urology Surgery, Children’s Hospital Affiliated to Shandong University, Jinan, Shandong, China

**Keywords:** CA-MRSA, proteomics, metabolomics, amino acid metabolism, lipid metabolism

## Abstract

**Objective:**

Bacterial culture and drug sensitivity testing have been the gold standard for confirming community-acquired methicillin-resistant *Staphylococcus aureus* (CA-MRSA) infection in breast abscess with a long history. However, these tests may delay treatment and increase the risk of nosocomial infections. To handle and improve this critical situation, this study aimed to explore biomarkers that could facilitate the rapid diagnosis of CA-MRSA infection.

**Methods:**

This study for the first time applied label-free quantitative proteomics and non-targeted metabonomics to identify potential differentially expressed proteins (DEPs) and differentially expressed metabolites (DEMs) in breast abscess infected with CA-MRSA compared to methicillin-susceptible *S. aureus* (MSSA). The two omics data were integrated and analyzed using bioinformatics, and the results were validated using Parallel Reaction Monitoring (PRM). Receiver operating characteristic (ROC) curves were generated to evaluate the predictive efficiency of the identified biomarkers for diagnosing CA-MRSA infection.

**Results:**

After using the above-mentioned strategies, 109 DEPs were identified, out of which 86 were upregulated and 23 were downregulated. Additionally, a total of 61 and 26 DEMs were initially screened in the positive and negative ion modes, respectively. A conjoint analysis indicated that the amino acid metabolism, glycosphingolipid biosynthesis, and glycerophospholipid metabolism pathways were co-enriched by the upstream DEPs and downstream DEMs, which may be involved in structuring the related network of CA-MRSA infection. Furthermore, three significant DEMs, namely, indole-3-acetic acid, L-(−)-methionine, and D-sedoheptulose 7-phosphate, displayed good discriminative abilities in early identification of CA-MRSA infection in ROC analysis.

**Conclusion:**

As there is limited high-quality evidence and multiple omics research in this field, the explored candidate biomarkers and pathways may provide new insights into the early diagnosis and drug resistance mechanisms of CA-MRSA infection in Chinese women.

## 1 Introduction

Lactational breast abscesses are crucial risk factors for early breastfeeding cessation in postpartum women and poor infant weight gain (Pevzner and Dahan, 2020). Bacterial infection is the main cause of lactational breast abscesses. Through the ruptured nipple, bacteria retrograde to the gland. Coupled with milk stagnation as a good culture medium, bacteria accelerate the reproduction, resulting in mastitis, and inflammation spreads further to form abscesses (Yin et al., 2020). *Staphylococcus aureus* is the most common pathogenic bacteria causing breast abscesses. Over the past decade, community-acquired methicillin-resistant *S. aureus* (CA-MRSA), also known as a superbug, has been on the rise, causing breast abscesses and soft tissue infections, accounting for 19.1%–60.0% of *S. aureus* infections (Boccaccio et al., 2014). CA-MRSA is characterized by high-level drug resistance and complex mechanisms, which can increase infection-caused mortality and prolong hospitalization time (Li Y. et al., 2021). Clinicians may need to intervene more actively for CA-MRSA than methicillin-susceptible *S. aureus* (MSSA) infection. So far, bacterial culture and drug sensitivity experiments have been the gold standard for diagnosing CA-MRSA infection. However, these methods are time consuming, and the results are easily affected by reagents, experimental operations, sample contamination, and other factors. The application of effective antibiotics is often delayed, which brings severe challenges to clinical treatment and nosocomial infection. Rapid diagnosis and correct management of CA-MRSA infection are essential to avoid complications (Krogerus et al., 2019). Thus, the exploration of more reliable biomarkers for early identification of CA-MRSA-infected breast abscesses is meaningful and urgent.

With the improvement of evidence-based medicine and big multi-omics data, the construction of an accurate inspection system for multiple molecular biomarkers for the early diagnosis and resistance mechanism of CA-MRSA infection would be a strategic breakthrough (Karczewski and Snyder, 2018). There is a general consensus in recent literature that quantitative proteomics could be widely used to identify expression profiles and signal pathways, screen biomarkers, monitor the changes in response to clinical therapeutics, and study protein interaction networks (Cao et al., 2021; Zhang et al., 2021; Dong et al., 2022). Parallel Reaction Monitoring (PRM), based on high-throughput and precision mass spectrometry, has been broadly used for subsequent quantitative verification of target proteins, replacing traditional Western blot technology. This makes the protein validation no longer subject to commercial antibodies, instead of being carried out on large-scale biological samples. Many researchers (Ni et al., 2021; You et al., 2022) combine label-free with PRM to take advantage of wide coverage, converting relative to absolute quantification, to achieve the selection of disease markers and the establishment of diagnostic models. In the process of genetic information transmission from upstream to downstream, metabolites can regulate protein interaction, modify enzyme activity, and stability. Integrating the analysis of proteomics and metabolomics to verify and complement each other, screening differential expressed proteins (DEPs) and differential expressed metabolites (DEMs) with the same change trend and co-participated pathways, are feasible research strategies to fix on potential markers and describe the regulation network of biological organisms (Chen F. et al., 2022; Schumacher-Schuh et al., 2022).

In this study, through the comprehensive proteomic and metabolomic profiling approach, we characterized the potential DEPs and DEMs between CA-MRSA and MSSA and probed into the inflammatory microenvironment features of CA-MRSA-infected breast abscess. The results will provide candidate biomarkers for CA-MRSA early diagnosis and provide clues for multidrug resistance mechanism and the identification of new therapeutic targets.

## 2 Methods

### 2.1 Clinical samples and workflow

This study enrolled 60 patients with clinical diagnosis of breast abscesses and confirmed CA-MRSA or MSSA infection by bacterial culture. The case group comprised 24 patients with CA-MRSA infection, while the control group included 36 patients with MSSA infection. The following selection criteria were used: (1) local redness, swelling, fever and pain of the breast, accompanied by a sense of fluctuation; (2) ultrasonographic examination of the lesion showing fluid anechoic areas; and (3) if the abscess is deep, skin redness and swelling are often not obvious; if antipyretics or antibiotics have been applied before hospital visit, the body temperature and white blood cell (WBC) count may not rise. The exclusion criteria were as follows: (1) complicated with other part infections, such as respiratory or urinary system; (2) polymicrobial infection of patients; (3) accompanied by major systemic diseases or tumors; and (4) immunocompromised individuals. The patients’ body mass index (BMI), delivery number, previous history of antibiotic therapy, temperature, WBC, neutrophil, red blood cell (RBC), hemoglobin (HB), platelet (PLT), C-reactive protein (CRP), and ultrasound features of breast abscesses were collected. T-test and chi-square test were used for statistical analysis, and p<0.05 indicated a statistically significant difference.

**Figure 1:**
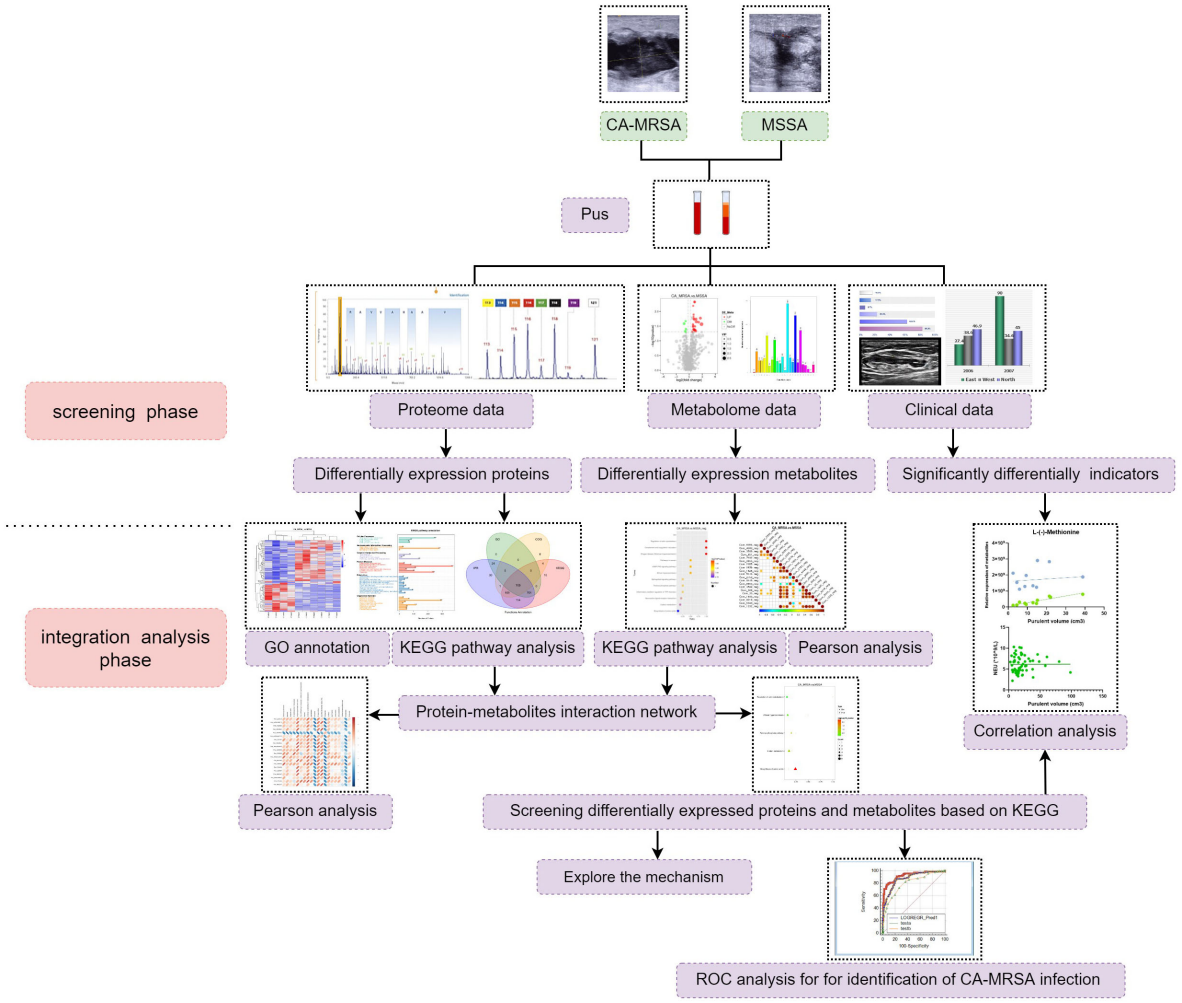
The research flow chart. A total of 60 patients with breast abscess were subsumed in this study, including 30 CA-MRSA as the case group and 36 MSSA as the control group. In the screening phase, label-free analysis, metabolomics analysis, and then PRM validation of candidate biomarkers were carried out. In the integration analysis phase, pathway enrichment, correlation analysis, and ROC analysis were also performed to further acquire insights from the omics and clinical data.

The CA-MRSA- or MSSA-infected pus samples were collected for the followed dual-omics studies. For label-free analysis, five CA-MRSA and five MSSA samples were used, followed by PRM validation of candidate biomarkers. In the metabolomics analysis, six samples were added to each group, respectively. Finally, the DEPs and DEMs closely related to CA-MRSA infection were screened from the conjoint analysis of proteomics and metabolomics (see **Figure 1**).

### 2.2 Label-free quantitative proteomic analysis

A total of 10 samples were minced individually with liquid nitrogen, pretreated with 2mM DL-dithiothreitol (DTT), and lysed in lysis buffer alkylated with indole acetic acid (IAA). Subsequently, the samples were centrifuged and the precipitates were collected, which dissociated in a dissolution buffer containing 0.1 M triethylammonium bicarbonate (TEAB, pH 8.5) and 8 M urea. Protein concentration was quantified by Bradford assay. The supernatant from each sample was digested with Trypsin Gold (Promega) at 1:50 enzyme-to-substrate ratio at 37°C for 16 h. The generated peptides were desalted with C18 cartridge to remove the high concentration urea and dried by vacuum centrifugation.

Proteomics analyses were performed using EASY-nLCTM 1200 UHPLC system (Thermo Fisher) coupled with a Q Exactive HF-X mass spectrometer (Thermo Fisher). All the resulting spectra were searched from the protein database by the search engine: Proteome Discoverer 2.2 (PD 2.2, Thermo). The protein quantification results were analyzed statistically using the t-test. The proteins that showed significant differences in quantification between the CA-MRSA and MSSA groups were considered as DEPs (p<0.05 and fold change (FC)>1.5 or FC<0.67). The DEPs were further categorized based on their molecular function (MF), cellular component (CC), and biological process (BP) for Gene Ontology (GO) mapping and annotation. Subsequently, the DEPs were searched against the Kyoto Encyclopedia of Genes and Genomes (KEGG) database (http://www.geneontology.org/) to obtain information on enriched functional pathways. Possible protein–protein interactions (PPIs) were predicted using the online STRING database (http://string-db.org/) and Cytoscape software.

### 2.3 Targeted validation by PRM

The DEPs that were significantly upregulated (FC>2.0) in the CA-MRSA group compared to the MSSA group were selected and were annotated as key proteins or pathways in GO and KEGG enrichment analysis. Using the peak area of the internal standard peptide segment DSPSAPVNVTV R labeled with heavy isotopes as the reference, the absolute quantification of target DEPs between CA-MRSA and MSSA groups were calculated in the PRM analysis after correction.

### 2.4 Untargeted metabolomics analysis (UHPLC-MS/MS)

The 22 pus samples were separately grinded with liquid nitrogen, and the homogenate was resuspended with prechilled 80% methanol and 0.1% formic acid by well vortexing. After centrifugation at 4°C, some of the supernatant was diluted to a final concentration containing 60% methanol by LC-MS grade water. The samples were subsequently transferred to fresh Eppendorf tubes with 0.22 μm filter. Finally, following centrifugation, the filtrate was injected into the LC-MS/MS system analysis.

LC-MS/MS analyses were performed using a Vanquish UHPLC system (Thermo Fisher) coupled with an Orbitrap Q Exactive series mass spectrometer (Thermo Fisher). The raw data files generated by super-high performance liquid chromatography-mass spectrum in series (UHPLC-MS/MS) were processed using the Compound Discoverer 3.1 (CD3.1, Thermo Fisher) to perform peak alignment and peak picking for each metabolite. Then, the peaks of metabolites were matched against the mzCloud (https://www.mzcloud.org/) and ChemSpider (http://www.chemspider.com/) databases to obtain the accurate and relative quantification. We performed multivariate statistical analysis using principal components analysis (PCA) and partial least squares discriminant analysis (PLS‐DA). Univariate analysis (t-test) was used to calculate the statistical significance (p-value). The metabolites with Variable Importance in the Projection (VIP) >1, p<0.05, and FC≥2/FC≤ 0.5 were deemed to be DEMs. Volcano plots were used to filter metabolites of interest, which were based on Log2 (FC) and -log10 (p-value) of metabolites. For clustering heat maps, the data were normalized using z-scores of the intensity areas of DEMs and were plotted by Python-3.5.0 R-3.4.3. We conducted Pearson correlation analysis to describe the relationship between DEMs. These DEMs were annotated using the KEGG database, HMDB database (http://www.hmdb.ca/), and Lipid Maps database (http://www.lipidmaps.org/). The metabolic pathways enriched by DEMs were considered statistically significant when p<0.05.

### 2.5 Integrated analysis of proteomics and metabolomics

The prominently screened DEPs and DEMs were measured for their degree of association based on the Pearson correlation coefficient, which ranges from -0.99 to +0.99. A negative correlation coefficient indicates negative correlation, while a positive correlation coefficient indicates positive correlation. All the DEPs and DEMs were mapped to the KEGG pathway database to obtain information on their common pathway enrichment.

### 2.6 Establishment of prediction model of CA-MRSA infection

We selected the DEMs in co-enrichment pathways of proteomics and metabolomics to predict CA-MRSA infection. The area under the curve (AUC) value of the receiver operating characteristic (ROC) was used to calculate the predictive efficiency of individual and combined DEMs. The association between the DEMs quantitation and purulent cavity volume was assessed using the Pearson correlation coefficient. Statistical analyses were performed using GraphPad Prism (version 9.0) and Stata (version 16.0). A p-value of <0.05 was considered statistically significant.

## 3 Result

### 3.1 Overview of the participants characteristics

**Table 1** presents the clinical characteristics of 24 breast abscesses with CA-MRSA and 36 with MSSA. The average age of the two groups was 29.08 ± 4.596 and 30.22 ± 5.060, respectively, without any confounding bias (p=0.3796). Compared to the MSSA group, the CA-MRSA group had a significantly lower NEU count (6.721 ± 1.847 vs. 5.254 ± 1.839×10^9^/L, p=0.0038), as shown in **Supplementary Figure S1A**, and a larger purulent cavity volume (15.85 ± 12.26 vs. 30.34 ± 21.70 cm^3^, p=0.0016) as shown in **Supplementary Figure S1B**. Additionally, no significant differences were found in the BMI, WBC, RBC, HB, PLT count, CRP, fever, reproductive number, and previous use of antibiotics between the two groups.

**Table 1:** Characteristics of study participants included in the CA-MRSA group and MSSA group.

	CA-MRSA (n=24)	MSSA (n=36)	p-value
Age	29.08 ± 4.596	30.22 ± 5.060	p=0.3796
BMI, kg/m^2^	25.97 ± 1.883	25.08 ± 2.407	p=0.1337
Delivery number			p=0.5936
1	13 (54.2)	22 (61.1)	
2	11 (45.8)	14 (38.9)	
Previous use of antibiotics			p=0.2057
Yes	14 (58.3)	15 (41.7)	
No	10 (41.7)	21 (58.3)	
WBC, ×10^9^/L	10.69 ± 3.059	11.93 ± 3.288	p=0.1458
NEU, ×10^9^/L	5.254 ± 1.839	6.721 ± 1.847	p=0.0038*
RBC, ×10^12^/L	4.071 ± 0.841	4.297 ± 0.783	p=0.2910
HB, g/L	130.7 ± 11.56	129.8 ± 12.94	p=0.7706
PLT, ×10^9^/L	209.8 ± 45.85	226.5 ± 50.05	p=0.1938
CRP, mg/L	21.15 ± 5.372	19.55 ± 3.960	p=0.1875
Fever			p=0.4095
Yes	18 (75.0)	23 (63.9)	
No	6 (25.0)	13 (36.1)	
Purulent cavity volume (cm3)	30.34 ± 21.70	15.85 ± 12.26	p=0.0016*
Purulent cavity characteristics			p=0.3293
Multiple	11 (45.8)	12 (33.3)	
Single	13 (54.2)	24 (66.7)	
Axillary fossa lymphadenectasis			p=0.4572
Yes	15 (62.5)	19 (52.8)	
No	9 (37.5)	17 (47.2)	

Data are expressed as the mean ± SD, or number of patients (percentages).

WBC, White Blood Cell; NEU, Neutrophil; RBC, Red Blood Cell; HB, Hemoglobin; PLT, Platelet, CRP, C-reactive Protein.

* Represents significant statistical difference.

From the perspective of ultrasonic images, the abscess cavity of CA-MRSA infection is characterized by thick walls and multiple compartments as shown in **Supplementary Figures S1C, D**. Concurrently, most patients have enlarged ipsilateral axillary lymph nodes accompanied by pressure pain. The structure of the axillary lymph node in the CA-MRSA group was disordered, with abundant blood flow signals according to Color Doppler Flow Imaging (CDFI). On the contrary, the structure in the MSSA group was relatively clearer, and there was sparser blood flow signal as shown in **Supplementary Figures S1E, F**.

### 3.2 Proteomic profiling and enriched pathways

**Figure 2:**
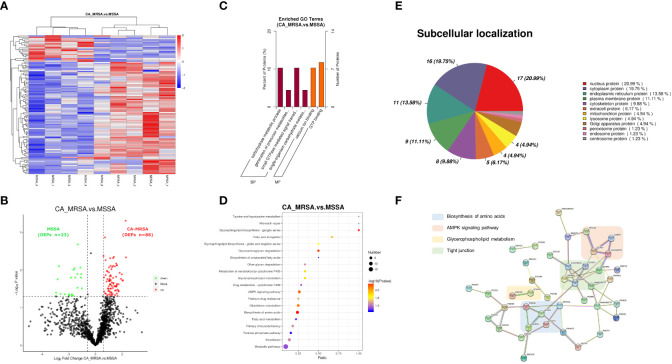
Proteomic profiling based on label-free between CA-MRSA and MSSA groups. **(A)** Hierarchical clustering analysis of heatmap in differentially expressed proteins (DEPs) between CA-MRSA and MSSA groups. Longitudinal axis is the clustering of samples, and horizontal axis is the clustering of proteins. Clustering of protein expression patterns among samples can be inferred from longitudinal clustering. Color depth from blue to red indicates the intensity detected by label-free LC-MS/MS quantification from low to high. **(B)** On the bases of the FC>1.5 and p-value<0.05 in the two groups, a volcano plot was mapped and used to show remarkable differences, which revealed 109 DEPs including 86 upregulations and 23 downregulations. Black indicates proteins with insignificant differences, red indicates upregulated proteins, and green indicates downregulated proteins. **(C)** GO significance analysis (p-value<0.05) can determine the main biological functions of DEPs. The percentage of ordinate represents x/n, x is the number of DEPs associated with this GO subtype, and n is the number of DEPs annotated by all GO. **(D)** The scatter plot of KEGG pathway enrichment analysis displays the top 20 pathways for DEPs enrichment in CA-MRSA group versus MSSA group. The X-axis shows the ratio of the number of DEPs to the total number of proteins identified in the corresponding pathway. The greater the value of abscissa, the higher the enrichment of differential proteins in this pathway. The redder the color, the smaller the p-value, and the more statistically significant. The larger the scatter, the more DEPs enriched in this pathway. **(E)** The proportion of the DEPs in each subcellular localization. **(F)** The protein–protein functional network crosslink of 39 DEPs was constructed using STRING database, and four functional clusters with CA-MRSA breast infection were formed.

The whole proteomic profiling identified a total of 1,363 proteins. A total of 109 DEPs (86 upregulated and 23 downregulated) between CA-MRSA and MSSA groups were revealed and displayed as a heatmap and volcano plot (**Figures 2A, B**). In the GO enrichment analysis, the DEPs were mainly involved in the carbohydrate metabolic process, generation of precursor metabolites and energy, calcium ion binding, and GTP binding in the categories of biological process and molecular function (**Figure 2C**). The top 20 pathways of DEPs enriched in the KEGG analysis are displayed in the scatter plot of **Figure 2D**. The first 10 pathways (p<0.05), considered significant, were mainly involved in the biosynthesis of amino acids, glycosphingolipid biosynthesis, glycosaminoglycan degradation, AMPK signaling pathway, glutathione metabolism, fatty acid elongation, and glycerophospholipid metabolism.

Each subcellular structure can provide a relatively independent place for a group of specific proteins to exercise their functions, and only proteins in the same or similar subcellular location will have interactions. The subcellular localization of the screened DEPs showed that nuclear proteins accounted for the highest proportion (20.99%), followed by cytoplasmic proteins (19.75%), endoplasmic reticulum proteins (13.58%), and plasma membrane protein (11.11%), respectively, as shown in **Figure 2E**. Defining the subcellular localization of the DEPs or expression products in the CA-MRSA group can provide the above directions for subsequent in-depth study of the mechanism. A total of 39 DEPs with significant enrichment variance between CA-MRSA and MSSA in GO and KEGG pathway analysis were selected to construct the interaction network. **Figure 2F** demonstrates the close relationships between four functional clusters with CA-MRSA infection: biosynthesis of amino acids, glycerophospholipid metabolism, AMPK signaling pathway, and tight junction. These clusters were seen to play crucial and interconnected roles in the occurrence of breast abscess infected by CA-MRSA.

### 3.3 PRM verification of candidate protein biomarkers

PRM was used to determine the abundance variation of 10 candidate DEPs (A0A140VK56, P07686, B2R983, Q5TEC6, Q53EU6, B4DVA7, V9HWE9, P00558, J3KPS3, and Q7L5N7) between CA-MRSA and MSSA groups. As illustrated in **Supplementary Figures S2A–J**, the expression levels of the above 10 candidate DEPs were increased in CA-MRSA versus MSSA (p<0.05). The relative expression of the target DEPs and peptides determined by label-free and PRM can be displayed in **Supplementary Table S1**, which indicates consistent upregulation trends (**Supplementary Figures S2K–T**).

### 3.4 Metabolomic profiling and related pathways

**Figure 3:**
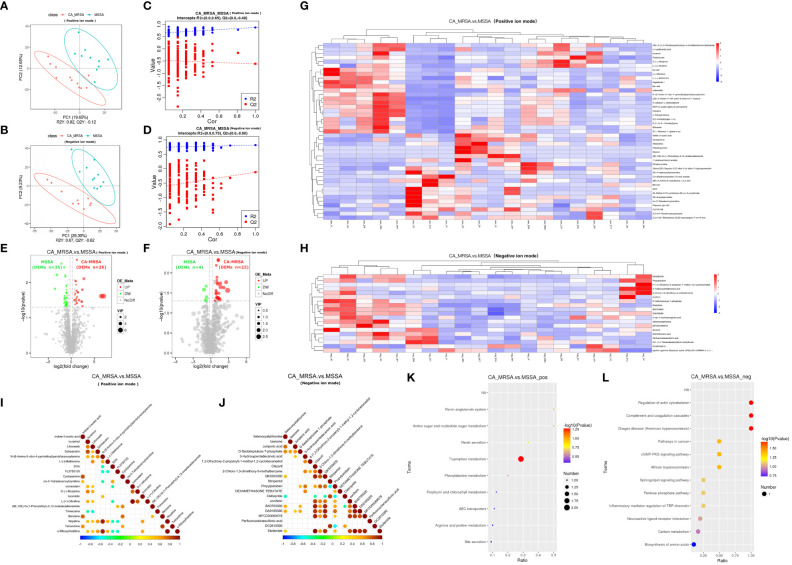
Comprehensive analysis of untargeted metabolomics of CA-MRSA and MSSA groups. **(A, B)** STRING PLS-DA score scatter plot in the positive ion mode (upper) and negative ion mode (lower), respectively. The abscissa is the score of the sample on the first principal component, and the ordinate is the score of the sample on the second principal component. R2Y indicates the interpretation rate of PLS-DA model, Q2Y is used to evaluate the prediction ability of PLS-DA model, and R2Y greater than Q2Y indicates that the model is well established. **(C, D)** Sequence verification diagram in the positive ion mode (upper) and negative ion mode (lower), respectively. The abscissa represents the correlation between the random grouping Y and the original grouping Y, and the ordinate represents the scores of R2 and Q2. When R2 data are greater than Q2 data and the intercept between Q2 regression line and Y-axis is <0, it indicates that PLS-DA model is accurate. **(E, F)** Volcanic map of DEMs in the positive ion mode (right) and negative ion mode, respectively (left). The DEMs whose expression increased in the CA-MRSA group are shown in red and those whose expression decreased are shown in green. In the positive ion mode, there are 61 DEMs screened with 26 upregulated and 35 downregulated. In the negative ion mode, there are 26 DEMs screened with 22 upregulated and 4 downregulated. **(G, H)** The clustering heat map of DEMs (the upper frame is positive ion mode, and the lower frame is negative ion mode). Longitudinal axis is the clustering of samples; lateral axis is the clustering of DEMs, the shorter clustering branch represents the higher similarity. Clustering of DEMs content between groups can be seen by horizontal comparison. **(I, J)** Correlation diagram of DEMs in the positive ion mode (right) and negative ion mode, respectively (left). The highest correlation was 1, indicating a complete consistent and synergistic correlation marked as red, and the lowest correlation was −1, indicating an opposite and mutually exclusive correlation marked as blue, and the parts without color indicate p-value >0.05. The figures show the linkage between the DEMs of Top 20 sorted by p-value from small to large. **(K, L)** The scatter plot of KEGG pathway enrichment analysis displays the top 20 pathways for DEMs enrichment in CA-MRSA group versus MSSA group. The greater the value of abscissa, the higher the enrichment of differential proteins in this pathway. The redder the color, the smaller the p-value, and the more statistically significant. The larger the scatter, the more the DEMs enriched in this pathway.

Because two samples in the CA-MRSA group originating from pus of the right and left breast abscesses in the same case, they were combined in metabolomics to be analyzed as one sample. In positive and negative ion patterns, the relational model between metabolite expression and group category (CA-MRSA and MSSA) was established by partial least squares-discriminate analysis (PLS-DA) (**Figures 3A–D**). The models provided 61 and 26 DEMs in the positive and negative ion mode (VIP>1.0 and p<0.05), respectively, including 48 upward and 39 downward. The volcano maps and heatmaps visually depicted the overall distribution of DEMs between CA-MRSA and MSSA groups detected in the positive and negative types (**Figures 3E–H**). As displayed in **Figures 3I, J**, in the positive ion mode, DEMs such as n-ribosylhistidine, N-(6-amino-5-nitro-4-pyrimidinyl), nopaline, GDP-N-acetyl-alpha-D-perosamine, and L-(-)-methionine had significant positive correlation with each other with the consistency of changing trends. Pathway enrichment analysis of the DEMs was statistically associated with the regulation of actin cytoskeleton, amino sugar and nucleotide sugar metabolism, complement and coagulation cascades, and cGMP-PKG signaling pathway in negative pattern (p<0.05). Other pathways in the positive pattern, such as the sphingolipid signaling pathway and tryptophan metabolism, failed to attain statistical significance, but their p-values were slightly >0.05, suggesting potential contributions as CA-MRSA-infected breast abscesses related metabolic pathways (**Figures 3K, L**).

### 3.5 Integrated network analysis of proteomic and metabolomic

**Figure 4:**
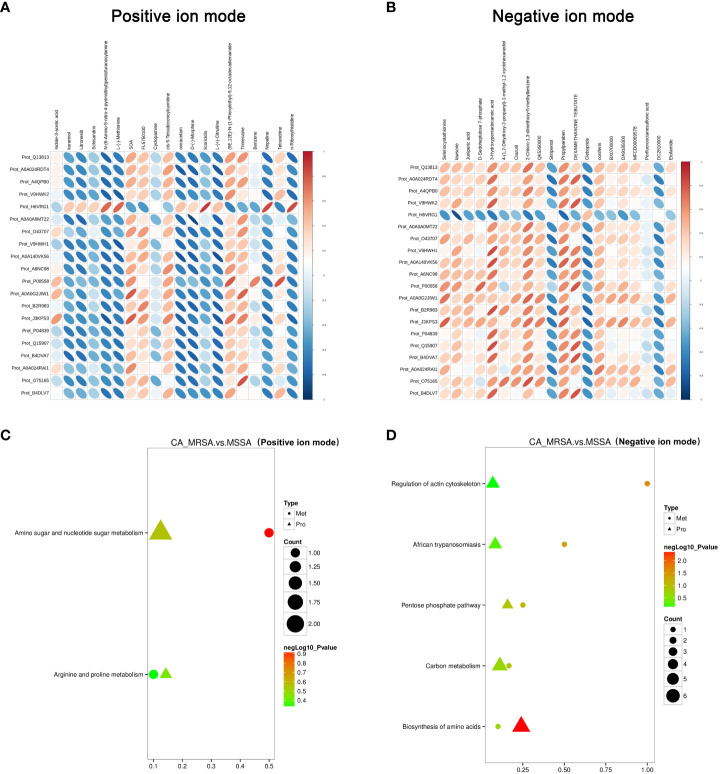
Conjoint pathway and network analysis of proteomic and metabolomic. **(A, B)** Relevance analysis between potential DEPs and DEMs based on Pearson correlation coefficient in the positive and negative ion mode, respectively. The horizontal axis represents the clustering of proteins, and the longitudinal axis represents the clustering of metabolites. The thicker and shorter the cluster branches, the stronger the correlation. The redder the cluster branches, the stronger the positive correlation. The bluer the cluster branches, the stronger the negative correlation. **(C, D)** Association analysis of KEGG pathway between potential DEPs and DEMs in the positive and negative ion mode, respectively. Proteins are represented by triangles and metabolism by dots. The closer the color is to red, the more significant the difference is in enrichment of this pathway. Count represents the ratio of the number of DEPs and DEMs in this pathway to the total number of proteins or metabolites identified in this pathway. The greater the value, the higher the enrichment degree of DEPs and DEMs in this pathway.

The critical DEPs and DEMs screened from proteomic and metabolomic analysis were applied to clarify the correlation degree and inter-reaction (**Supplementary Table S2**). In **Figures 4A, B**, the DEPs of A0A140VK56, P00558, J3KPS3, and B4DVA7 were consistent with bradykinin, 3-hydroxypentadecanoic acid, and D-sedoheptulose 7-phosphate, involve in the regulation of actin cytoskeleton, biosynthesis of amino acids, sphingolipid signaling pathway, and pentose phosphate pathway in negative mode. Furthermore, the above-mentioned DEPs were relevant to nopaline, L-(-)-methionine, indole-3-acetic acid, and n-ribosylhistidine in terms of the amino acid metabolism comprised of amino sugar and nucleotide sugar metabolism, arginine and proline metabolism, and tryptophan metabolism in positive mode (**Figures 4C, D**).

### 3.6 Identification of CA-MRSA infection and correlation analysis

**Figure 5:**
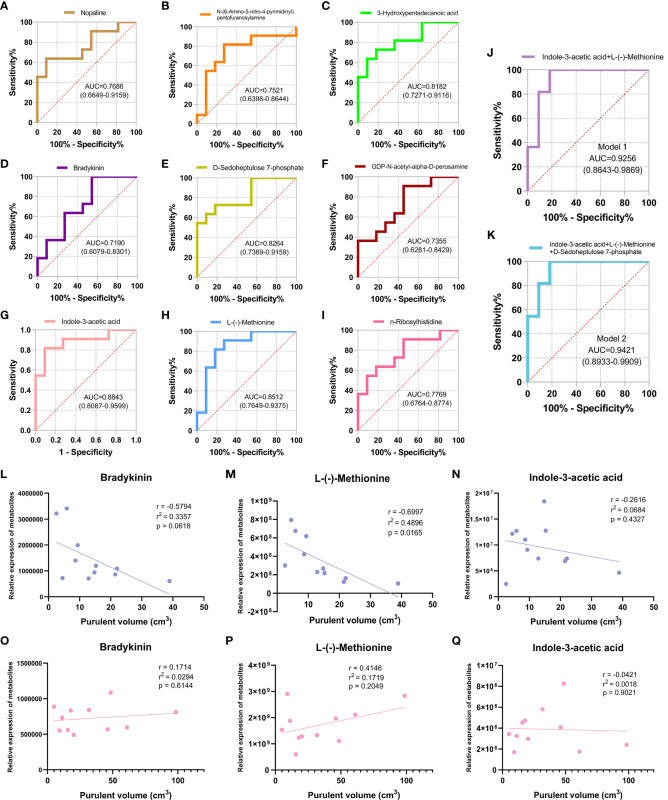
Identification of CA-MRSA infection with ROC and clinical correlation analysis of the candidate DEMs. **(A-I)** Selected nine candidate DEMs (bradykinin, 3-hydroxypentadecanoic acid, D-sedoheptulose 7-phosphate, nopaline, L-(-)-methionine, n-ribosylhistidine, indole-3-acetic acid, N-(6-amino-5-nitro-4-pyrimidinyl), and GDP-N-acetyl-alpha-D-perosamine) to draw ROC curve. The area under curve (AUC) values and 95% confidence intervals (CIs) are listed in the ROC diagram for each candidate metabolite to assess the diagnostic value of CA-MRSA infection. **(J)** Model 1 of the two metabolites indole-3-acetic acid and L-(-)-methionine showed a good discriminative ability to identify CA-MRSA infection (AUC, 0.9256; 95% CI, 0.8643–0.9869). **(K)** Model 2 of the three metabolites indole-3-acetic acid, L-(-)-methionine, and D-sedoheptulose 7-phosphate showed a better recognition rate of CA-MRSA infection (AUC, 0.9421; 95% CI, 0.8933–0.9909). **(L-Q)** Scatter plot presents the three metabolites in Model 2 applied to establish the correlation analyses with purulent cavity volume. The blue scatter plot represents the CA-MRSA group, and the pink scatter plot represents the MSSA group. Related coefficient r, linear regression r^2^ values, and p-values are listed.

The ROC curve was plotted for the nine screened DEMs, and the AUC value was used to assess the diagnostic accuracy of individual DEMs for CA-MRSA infection (**Figures 5A–I**). To better define the optimal diagnostic model, we performed an ROC analysis for a set of biomarkers, including indole-3-acetic acid, L-(-)-methionine, and D-sedoheptulose 7-phosphate, whose AUC values were the top 3. As presented in **Figures 5J, K**, Model 2 of the mentioned three metabolites showed the best recognition rate of CA-MRSA infection (AUC value, 0.9421; 95% CI, 0.8933–0.9909) compared with Model 1. Furthermore, we selected the three metabolites in Model 2 to continue the association analysis with purulent cavity volume (**Figures 5L–Q**). The results showed that only the content of L-(-)-methionine in the CA-MRSA group was negatively correlated with purulent cavity volume, but there were no correlations in the MSSA group or other DEMs.

## 4 Discussion

To the best of our knowledge, this research is the first attempt to link proteomics with metabolomics to investigate the biomarkers and related mechanisms of CA-MRSA infection. The integrated analysis elaborated the pathways of amino acid metabolism and lipid metabolism co-enriched by upstream DEPs and downstream DEMs, which may be involved in structuring the related network of breast abscess infected by CA-MRSA in Chinese women.

### 4.1 Amino acid metabolism

**Figure 6:**
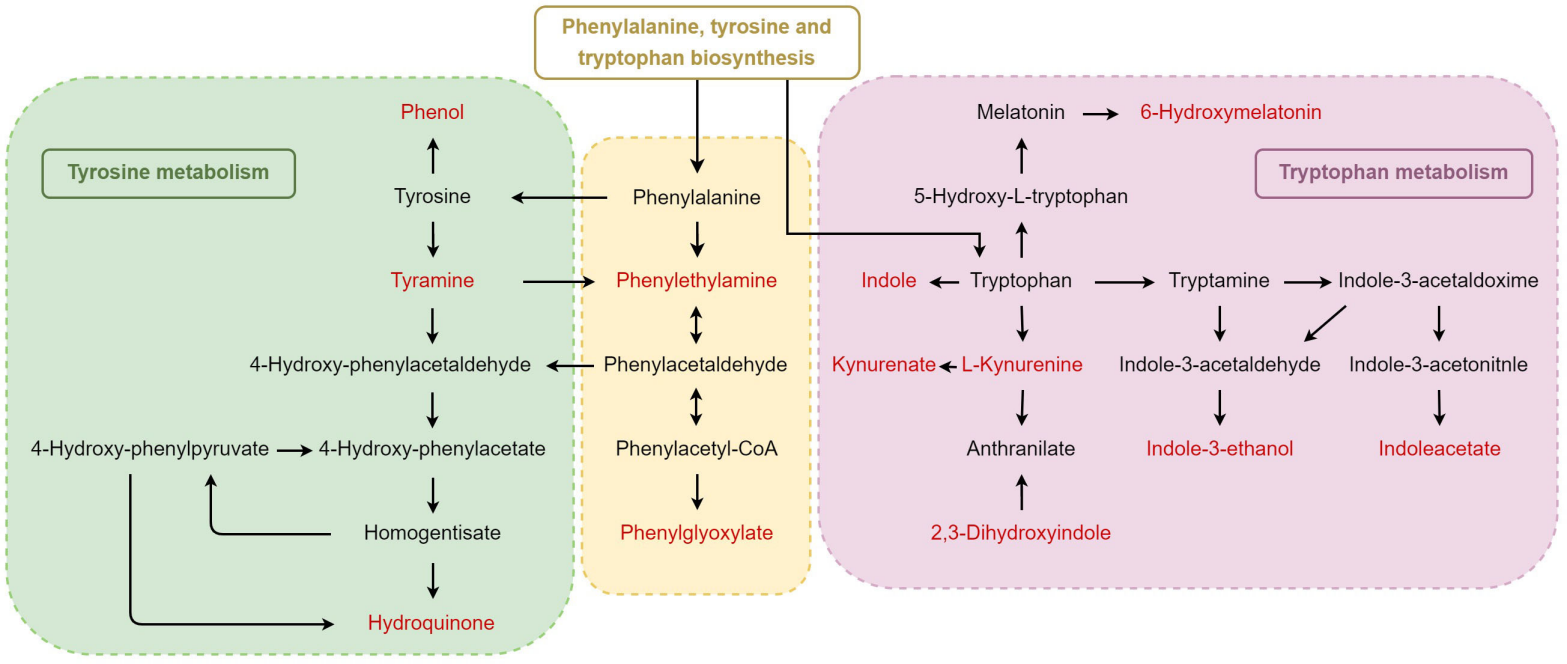
Schematic diagram of the crucial pathways of amino acid metabolism. Phenylalanine, tyrosine and tryptophan biosynthesis, tyrosine metabolism, and tryptophan metabolism were the top hits from the pathway analysis of CA-MRSA-specific metabolites. The upregulated metabolites in CA-MRSA infection are marked red compared with the MSSA group.

Amino acids are the basic units of proteins. The amino acid metabolites of microorganisms and host cells are widely implicated in biological processes, such as inflammation and immune response, by regulating the activation of immune cells and the production of antibodies (Costantini et al., 2020; Dhankhar et al., 2020). These have played essential roles in adaptive and innate immunity. Recent literature has documented the important intermediate metabolites in the aromatic amino-acid-related pathways that undergo various changes in sepsis (Chen Q. et al., 2022). The catabolic pathway of converting phenylalanine to phenylpyruvate can impel the escape state of neutrophils in *Acinetobacter baumannii* (Rodman et al., 2019). In our study, the DEPs between CA-MRSA and MSSA are mostly related to kinases or transferases in the amino acid metabolic pathways. By regulating the synthesis or catabolism of amino acids, the expressions of downstream DEMs are affected correspondingly, leading to the most prominent and widespread alterations in amino acid biosynthesis, phenylalanine metabolism, tryptophan metabolism, arginine, and proline metabolism in the CA-MRSA group (**Figure 6**). On the other hand, biofilm is an important pathogenic and drug-resistant cause of CA-MRSA. It not only helps bacteria colonize in the host tissue and spreads toxins *in vivo* but also provides physical and chemical protective barriers for bacterial survival and reinforces the resistance of bacteria to antibiotics and host immune response clearance. Research has announced that transformations in the microenvironment of L-tyrosine residues in phenylalanine metabolism can cause disturbance of cell wall peptidoglycan synthesis and membrane protein conformation in biofilms of CA-MRSA (Zhang et al., 2022).

Tryptophan (Trp) catabolism can lead to Trp depletion and the accumulation of related metabolites, thereby mediating immune escape. Their influences on the host and microorganisms can be achieved through two different catabolic pathways: (1) approximately 90%–95% of Trp is converted into kynurenine (Kyn) and downstream metabolites via the Kyn pathway (Dehhaghi et al., 2019). (2) Approximately 4%–6% of unabsorbed Trp is metabolized by microorganisms into indole and indolic compounds (Wyatt and Greathouse, 2021), consisting of indole-3-acetic acid (IAA), indole ethanol, and indole-3-propionic acid (IPA) (Li J. et al., 2021). Under the catalysis of tryptophan monooxygenase and indole-3-acetamide hydrolase, microorganisms can decompose tryptophan to IAA. An *in vivo* study substantiated that IAA is beneficial in ameliorating hepatic inflammation that presents in non-alcoholic fatty liver disease (NAFLD) mice, which may be relevant to the lightened inflammatory responses mediated by macrophages (Ji et al., 2019). Mice fed with a high-fat diet (HFD) were infected by MRSA, whose tryptophan metabolite IAA was dramatically reduced, resulting in insensitivity to antibiotic treatment, while combined usage of IAA and ciprofloxacin increased the survival rate for mice (Liu et al., 2021). Similarly, our finding has linked the abnormal metabolism of IAA to CA-MRSA infection and drug resistance, using IAA as one of the potential biomarkers for early diagnosis. More research should decipher the specific mechanisms behind the changes of IAA levels in CA-MRSA infection.

Furthermore, a low expression of L-methionine was found in the CA-MRSA group, and the level was negatively correlated with the volume of breast abscess. Methionine is the most significant methyl donor in the body. By methylating glutathione peroxidase (GPX) and superoxide dismutase (SOD), methionine could increase the synthesis of endogenous phospholipids, thereby stabilizing the lysosomal membrane and reducing the release of acid phosphatase. Thus, a deficiency of methionine can cause local inflammation that is not easily limited and subsequently spreads rapidly. Additionally, L-methionine has the ability to stimulate T-cell activation and maintain T-cell survival and function. L-Methionine intake increases during T-cell activation and contributes to the production of S-adenosine methionine (SAM), which maintains histone and RNA methylation (Sinclair et al., 2019; Bian et al., 2020). However, L-methionine starvation can decrease H3K79me2 in CD8+T cells (Bian et al., 2020) and H3K4me3 in Th17 cells (Roy et al., 2020), thereby limiting the survival and function of T cells. Therefore, it is speculated that the reduced expression of L-methionine in the CA-MRSA group is a potential mechanism for local inflammatory reaction and immune suppression after CA-MRSA infection, which requires further discussion and elucidation.

### 4.2 Glycosphingolipid biosynthesis and glycerophospholipid metabolism

Glycosphingolipids (GSLs) are binding lipids composed of ceramide and oligosaccharide chains. They are common components of the eukaryotic cell plasma membrane, extending their polar end of hydrophilic sugar chains outward, thus becoming a sign of cell surface bioactivity. The synthesis of GSLs originates from the meshwork of vesicles and tubules in the smooth endoplasmic reticulum (ER) and completes in the Golgi apparatus (Rizzo et al., 2021), where they modulate the activities of membrane proteins, especially signaling receptors. Therefore, GSLs play an important role in molecular signal transduction, immune response, cell adhesion, and recognition (Zhang et al., 2019). Based on the presence of sialic acid, GSLs are divided into neutral and acidic, with the latter known as gangliosides (Chiricozzi et al., 2021). In our data, the pathway of GSLs biosynthesis-ganglio series, in which the DEPs (ID: P07686, B4DVA7) participate, was markedly enriched in the CA-MRSA group (p=0.003). Meanwhile, the DEPs enriched in glycosphingolipids biosynthesis of the CA-MRSA group was localized in subcellular structures such as the endoplasmic reticulum and Golgi apparatus, which, to some extent, supports the subcellular localization finding that larger proportions of DEPs in the CA-MRSA group belonged to cytoplasmic proteins (19.75%), endoplasmic reticulum proteins (13.58%), and plasma membrane proteins (11.11%). The protein annotation of P07686 and B4DVA7 is both β-hexosaminidase B (HexB), which is a group of the most active glycosyl hydrolase isozymes in the lysosomes. HexB catalyzes the conversion from GM2 to GM3 in ganglioside biosynthesis. Substrates for HexB are acidic GSLs, which are implicated in specific infection. Acidic GSLs sugar chains on the host cell surface are binding receptors for many bacterial toxins and determinants of cellular recognition (Aerts et al., 2019). Since an intensified inflammatory state is marked by an increase in the activity of lysosomal exo-glycosidases in tissues and body fluids, especially the most active HexB (Chojnowska et al., 2018), we propose a hypothesis that the combination of CA-MRSA pathogen and GSLs activates HexB with the local inflammatory response to breast abscesses, which is critical for GSLs synthesis and mediates the specific infection process caused by CA-MRSA.

Our findings suggest that lysophosphatidylcholine acyltransferase 2 (LPCAT2, ID: Q7L5N7) is involved in the glycerophospholipid (GPL) metabolism pathway and is significantly enriched in the CA-MRSA breast abscess group (p=0.036). Abnormal GPL metabolism, represented by phosphatidylcholine (PC), has been confirmed to play a considerable role in inflammation, hypertension, and tumors (Sonkar et al., 2019; Sun et al., 2021; Chen Q. et al., 2022). PC is an important component of the biofilm structure. LPCAT2 can acylate lysophosphatidylcholine (LPC) to form PC, while the main source of LPC is the cleavage of PC by phospholipase A2 (PLA2), and the two routes are mutually interconverted (Liu et al., 2020). Recent evidence suggests that LPC possesses advantageous bactericidal clearance and anti-inflammatory effects by enhancing the permeability and perturbation to the cell membrane of CA-MRSA (Miyazaki et al., 2017). LPC reinforces the sensitivity of multidrug-resistant *Acinetobacter baumannii* to colistin (Miró-Canturri et al., 2021) and strengthens the bactericidal activity of neutrophils by intensifying H_2_O_2_ production. In *Mycobacterium tuberculosis* infection, LPC also restrains the inflammatory cytokines production in macrophages via the cAMP-activated PKA-PI3K-P38 MAPK signaling pathway, which can alleviate excessive inflammation (Lee et al., 2018). As the key enzyme in the transition from LPC to PC, high horizontal LPCAT2 in the CA-MRSA group decreased the expression of LPC and weakened its anti-inflammatory action. The lowered LPC/PC ratios are associated with the severity of the inflammatory process (Boldyreva et al., 2021). The above-mentioned may be the clues for CA-MRSA breast abscesses appearing more severe inflammatory response and insensitivity to antibiotics. In addition, LPCAT2 is highly expressed in inflammatory cells and is thought to contribute to the production of lipid mediators in these cells (Tsoupras et al., 2018). LPCAT2 participates in PC remodeling activity in lipid droplet (LD) (Cotte et al., 2018; Jiang et al., 2019) and acts as a key enzyme in the Lands’ Cycle pathway to synthesize PC (Wang and Tontonoz, 2019), which may also affect the plasma membrane composition and the drugs’ pharmacokinetics. Alterations in lipid composition between sensitive and resistant bacteria lead to ineffective drug treatment based on liposome delivery (Saito et al., 2022). In summary, it can be inferred that the resistance of CA-MRSA may be well correlated with the change in plasma membrane structure. This could constitute future directions for untangling the intricate mechanisms of LPCAT2-mediated CA-MRSA inflammation and drug resistance.

### 4.3 Limitation

Linking proteomics with metabolomics to probe into the biomarkers and related mechanisms of CA-MRSA infection and the biological microenvironment characteristics of CA-MRSA infection in breast abscess were described from the perspectives of amino acid metabolism and lipid metabolism. For all that, several limitations should be acknowledged. First, our sample size was relatively small so as to be considered exploratory until verified in subsequent large cohort studies. From another viewpoint, the small sample size can still provide statistical confidence for our results due to substantial changes in metabolites that could be easily measured. Second, it would be more understandable and useful if we perform follow-up metabolomics profiling of the isolated MRSA and MSSA to validate our findings. Third, the limited number of potential metabolites screened hindered our further analysis using the machine learning methods.

### 4.4 Conclusion

This research is the first to describe the proteomic and metabolomic profiles of clinically well-characterized breast abscesses caused by CA-MRSA and MSSA in Chinese women. Most of the DEPs and DEMs associated with CA-MRSA infection were found to be enriched in amino acid metabolism, glycosphingolipid biosynthesis, and glycerophospholipid metabolism pathways. More importantly, indole-3-acetic acid, L-(−)-methionine, and D-sedoheptulose 7-phosphate showed good discriminative abilities in identifying early CA-MRSA infection of breast abscesses in a clinical setting. Given the limited high-quality evidence and multi-omics studies in this field, more insights and benefits are required to elucidate the molecular mechanisms of potential biomarkers and pathways for early diagnosis of CA-MRSA infection and drug resistance in the future.

## Data availability statement

The data presented in the study are deposited to the ProteomeXchange Consortium via the PRIDE partner repository with the dataset identifier PXD046678.

## Ethics statement

The study was approved by scientific and ethical review (No. SDTHEC202100318) and conforms to the principles in the Declaration of Helsinki. The patients included were informed and agreed to undergo the examination, contribute their demographic and clinical information, and donate their pus specimens for laboratory research.

## Author contributions

Conceptual design, YY and XG; Methodology, YL, ZL, and QM; Validation, MZ and CS; Bioinformatics analysis, XG and LC; Data curation, CL; Writing—original draft preparation, YY; Writing—review and editing, XG; Supervision, ZY; Funding acquisition, XG and YY. All authors contributed to the article and approved the submitted version.

## Supplementary material

Supplementary Figure 1 Clinical indicators with statistical differences and ultrasonographic signs between CA-MRSA and MSSA groups. **(A)** The NEU count was obviously lower in CA-MRSA group comparing with MSSA group (p=0.0038). **(B)** The CA-MRSA group have larger purulent cavity volume than the MSSA group (p=0.0016). **(C, D)** From the perspective of ultrasonic imaging features, the breast abscess cavity formed by CA-MRSA infection is comparatively greater, and the ultrasonic signs show the cyst wall thickness and cyst cavity multiple compartments. **(E, F)** The structure of the ipsilateral axillary lymph node in CA-MRSA group was disordered, where the blood flow signals were abundant according to Color Doppler Flow Imaging (CDFI). The structure of the ipsilateral axillary lymph node in MSSA group is relatively clear, and there is no obvious blood flow signal in way of CDFI.

Supplementary Figure 2 Parallel reaction monitoring (PRM) verification of candidate DEPs. **(A-J)** Select 10 candidate DEPs (A0A140VK56, P07686, B2R983, Q5TEC6, Q53EU6, B4DVA7, V9HWE9, J3KPS3, P00558 and Q7L5N7) of pus samples between CA-MRSA and MSSA groups, according to the chromatographic peak area of the target peptide, the relative expression amount of the target protein in each sample is obtained. In each target protein, the left three columns are CA-MRSA infected breast abscess samples, and the right three columns are MSSA infected breast abscess samples. **(K-T)** The relative expression of the 10 DEPs determined by label-free and PRM can be displayed with orange and green separately, which indicate consistent up-regulation trends in the two ways.
