# Supplementary figures and images for: Integrative proteomic and metabonomic profiling elucidates amino acid and lipid metabolism disorder in CA-MRSA-infected breast abscesses
Source: Front Cell Infect Microbiol. 2023 Nov 13;13:1240743. doi: 10.3389/fcimb.2023.1240743 (PMC10679464; doi:10.3389/fcimb.2023.1240743)

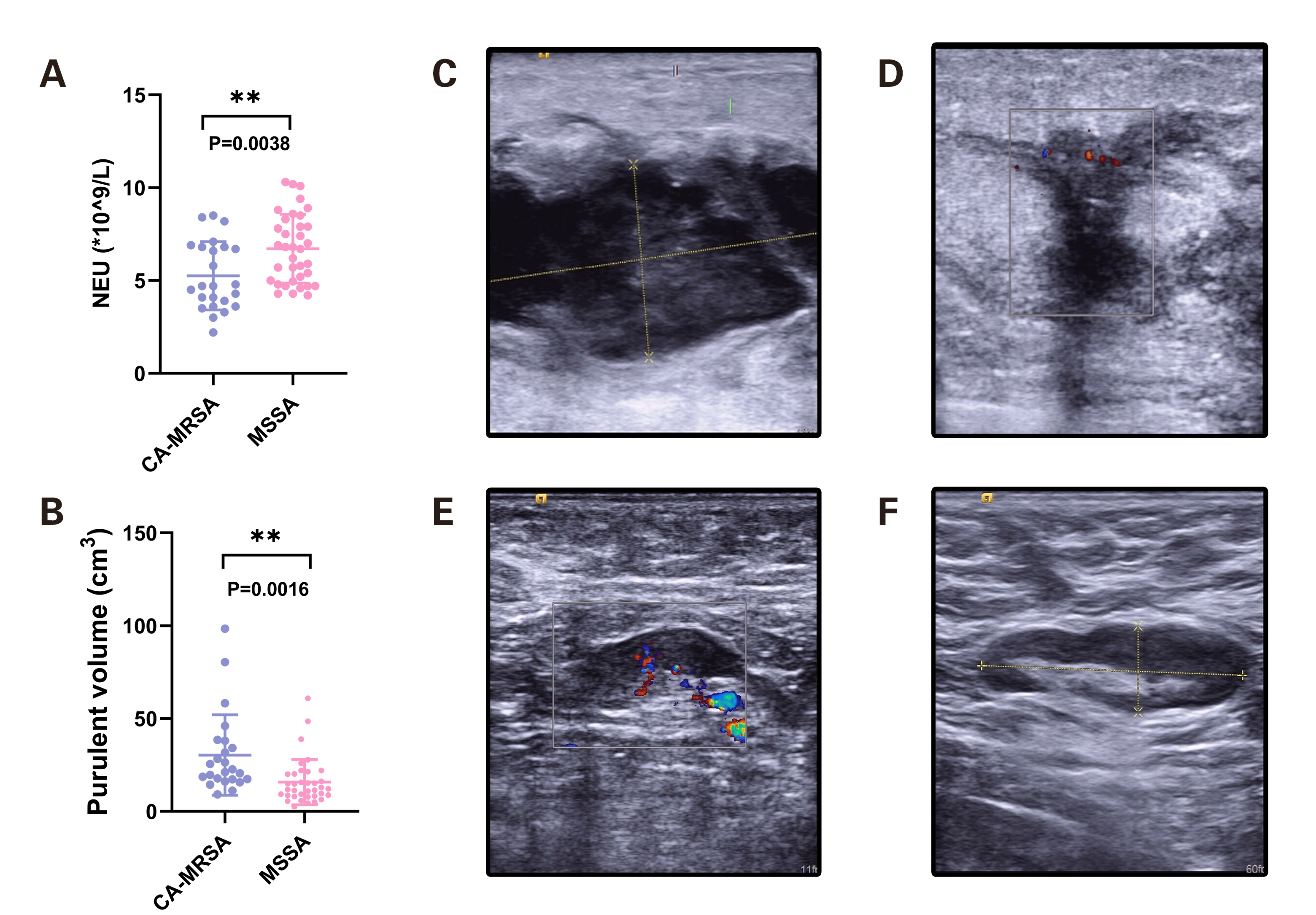

Supplement: Supplementary Figure 1 — Clinical indicators with statistical differences and ultrasonographic signs between CA-MRSA and MSSA groups. (A) The NEU count was obviously lower in CA-MRSA group comparing with MSSA group (p=0.0038). (B) The CA-MRSA group have larger purulent cavity volume than the MSSA group (p=0.0016). (C, D) From the perspective of ultrasonic imaging features, the breast abscess cavity formed by CA-MRSA infection is comparatively greater, and the ultrasonic signs show the cyst wall thickness and cyst cavity multiple compartments. (E, F) The structure of the ipsilateral axillary lymph node in CA-MRSA group was disordered, where the blood flow signals were abundant according to Color Doppler Flow Imaging (CDFI). The structure of the ipsilateral axillary lymph node in MSSA group is relatively clear, and there is no obvious blood flow signal in way of CDFI. [file Image_1.jpeg]

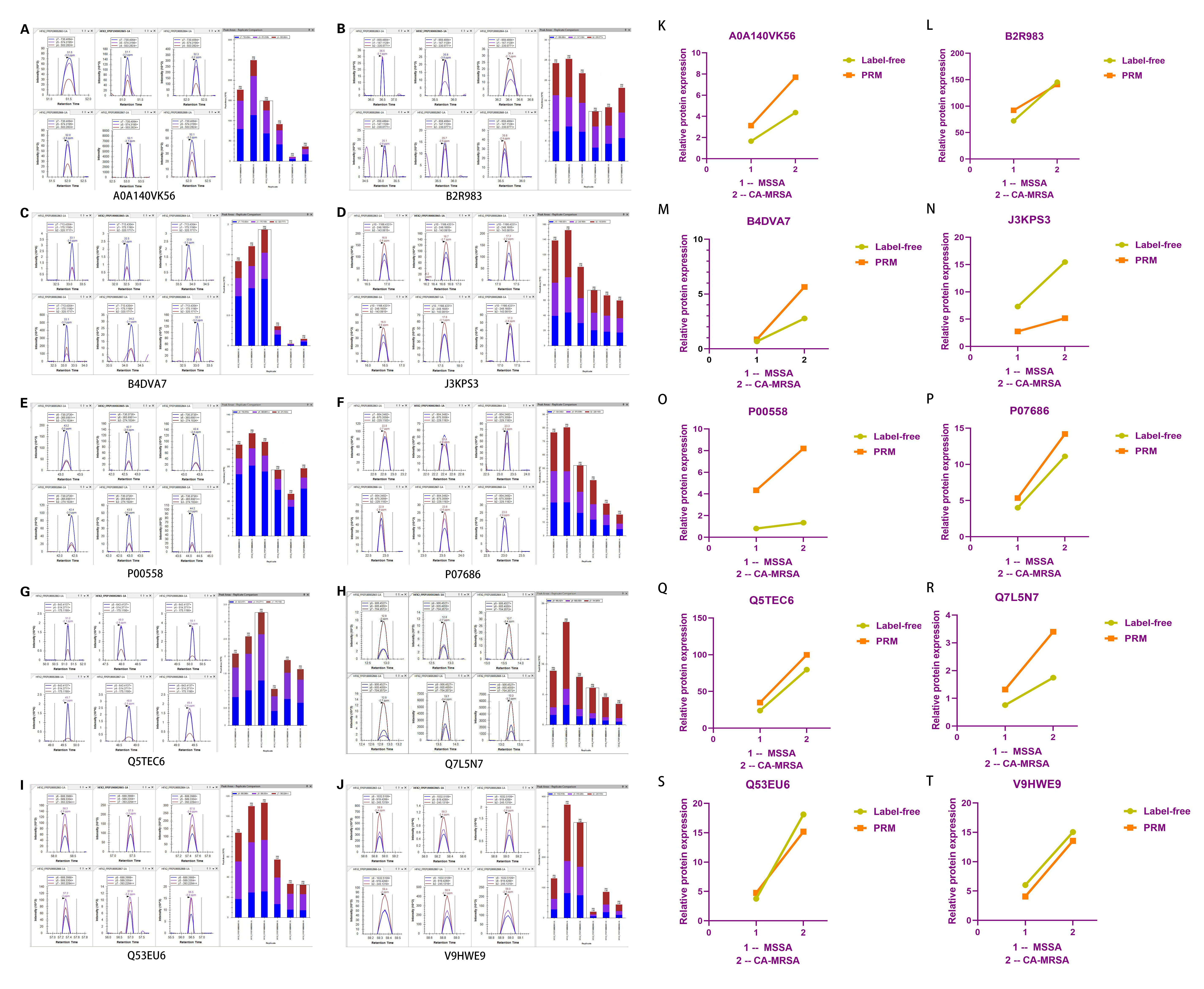

Supplement: Supplementary Figure 2 — Parallel reaction monitoring (PRM) verification of candidate DEPs. (A-J) Select 10 candidate DEPs (A0A140VK56, P07686, B2R983, Q5TEC6, Q53EU6, B4DVA7, V9HWE9, J3KPS3, P00558 and Q7L5N7) of pus samples between CA-MRSA and MSSA groups, according to the chromatographic peak area of the target peptide, the relative expression amount of the target protein in each sample is obtained. In each target protein, the left three columns are CA-MRSA infected breast abscess samples, and the right three columns are MSSA infected breast abscess samples. (K-T) The relative expression of the 10 DEPs determined by label-free and PRM can be displayed with orange and green separately, which indicate consistent up-regulation trends in the two ways. [file Image_2.jpeg]
