# Supplementary material for: Integrative proteomic and metabonomic profiling elucidates amino acid and lipid metabolism disorder in CA-MRSA-infected breast abscesses
Source: Front Cell Infect Microbiol. 2023 Nov 13;13:1240743. doi: 10.3389/fcimb.2023.1240743 (PMC10679464; doi:10.3389/fcimb.2023.1240743)
Supplement: Supplementary file 3 [file Table_1.docx]

**Supplementary Table 1 The validation of target differentially expressed proteins and peptides with PRM**

| Target DEPs^a^ | Label-free | | Target peptides | PRM | | Genes | Description |
| --- | --- | --- | --- | --- | --- | --- | --- |
|  | FC^b^ | p-value |  | FC | p-value |  |  |
| A0A140VK56 | 2.6176 | 0.0045 | IEDGNNFGVAVQEK | 2.4928 | 0.0071 | TALDO1 | Transaldolase |
| B4DVA7 | 3.0529 | 0.0234 | YRPGTVALR | 6.5006 | 0.0028 | HEXB | Beta-hexosaminidase |
| Q53EU6 | 4.7918 | 0.0005 | LPVSDVLR | 3.1838 | 0.0025 | GPAT3 | Glycerol-3-phosphate acyltransferase 3 |
| Q5TEC6 | 3.3302 | 0.0240 | STELLIR | 2.8603 | 0.0108 | HIST2H3PS2 | Histone H3 |
| P07686 | 2.7712 | 0.0466 | EVVEEAENGR | 2.6572 | 0.0116 | HEXB | Beta-hexosaminidase subunit beta |
| B2R983 | 2.0159 | 0.0116 | DNSGMIDKNELK | 1.5284 | 0.0462 | GSTO1 | cDNA, FLJ94267, highly similar to Homo sapiens glutathione S-transferase omega 1 (GSTO1), mRNA |
| J3KPS3 | 2.1137 | 0.0147 | AAEDDEDDDVDTK | 1.8699 | 0.0089 | ALDOA | Fructose-bisphosphate aldolase, class-I |
| V9HWE9 | 2.4932 | 0.0163 | MLLADQGQSWK | 3.3215 | 0.0028 | HEL-S-22 | Epididymis secretory protein Li 22 |
| P00558 | 1.6568 | 0.0421 | DSAYPEELSR | 1.8872 | 0.0043 | PGK1 | Phosphoglycerate kinase 1 |
| Q7L5N7 | 2.2971 | 0.0390 | EVQTTPSTASNK | 2.5808 | 0.0001 | LPCAT2 | Lysophosphatidylcholine acyltransferase 2 |

Based on the establishment of biomarkers for CA-MRSA infected mammary abscess, 10 selected up-regulated proteins with differentially expressed for subsequent PRM validation

1. DEPs: differentially expressed proteins
2. FC refers to the fold-change of CA-MRSA vs MSSA
