# Supplementary material for: Integrative proteomic and metabonomic profiling elucidates amino acid and lipid metabolism disorder in CA-MRSA-infected breast abscesses
Source: Front Cell Infect Microbiol. 2023 Nov 13;13:1240743. doi: 10.3389/fcimb.2023.1240743 (PMC10679464; doi:10.3389/fcimb.2023.1240743)
Supplement: Supplementary file 4 [file Table_2.docx]

**Supplementary Table 2 The Relevance-Based Study between differential proteins and metabolites**

|  | Metabolites in positive mode r (p value) | | | | Metabolites in negative mode r (p value) | | |  |
| --- | --- | --- | --- | --- | --- | --- | --- | --- |
| Proteins | L-(-)-Methionine | Nopaline | n-Ribosylhistidine | Indole-3-acetic acid | 3-Hydroxypentadecanoic acid | D-Sedoheptulose 7-phosphate | Bradykinin |  |
| A0A140VK56 | -0.7817 (0.0128) | -0.6899 (0.0397) | -0.6871 (0.0409) | 0.7420 (0.0221) | 0.8281 (0.0058) | 0.6819 (0.0431) | 0.7633 (0.0167) | |
| P00558 | -0.6250(0.0719) | -0.6846 (0.0419) | -0.6791 (0.0443) | 0.6706 (0.0481) | 0.4827 (0.1881) | 0.4846 (0.1861) | 0.6349 (0.0662) | |
| J3KPS3 | -0.6552 (0.0554) | -0.7718 (0.0148) | -0.7607 (0.0173) | 0.8225 (0.0065) | 0.4682 (0.2037) | 0.7121 (0.0314) | 0.7549 (0.0187) | |
| B4DVA7 | -0.7003 (0.0357) | -0.5096 (0.1611) | -0.5118 (0.1591) | 0.5775 (0.1035) | 0.7486 (0.0203) | 0.6417 (0.0624) | 0.6403 (0.0632) | |

Data were presented as correlation coefficient (r) and p value.
